# Exploring the Role of Heat Shock Proteins in Neuroimmune Modulation in Rheumatoid Arthritis: Insights from a Rat Model

**DOI:** 10.3390/ijms26199743

**Published:** 2025-10-07

**Authors:** Malak Fouani, Federica Scalia, Giuseppe Donato Mangano, Francesca Rappa, Wassim Abou-Kheir, Angelo Leone, Nada Lawand, Rosario Barone

**Affiliations:** 1 Department of Neurology, Duke University, Durham, NC 27710, USA; malak.fouani@duke.edu; 2 Department of Biomedicine, Neurosciences and Advanced Diagnostics, University of Palermo, 90127 Palermo, Italy; federica.scalia@unikore.it (F.S.); francesca.rappa@unipa.it (F.R.); angelo.leone@unipa.it (A.L.); 3 Department of Medicine and Surgery, University of Enna “Kore”, 94100 Enna, Italy; giuseppe.mangano@unikore.it; 4 Department of Anatomy, Cell Biology and Physiological Sciences, Faculty of Medicine, American University of Beirut, Beirut 1107 2020, Lebanon; wa12@aub.edu.lb; 5 Ivy Tech Community College, Indianapolis, IN 46208, USA; nlawand@ivytech.edu

**Keywords:** rheumatoid arthritis (RA), heat shock proteins (HSPs), N-methyl-D-aspartate receptor (NMDAR), spinal cord, synovial membrane, chronic inflammatory autoimmune disease

## Abstract

Rheumatoid arthritis (RA) is a chronic inflammatory autoimmune disease affecting the joints, with neurogenic inflammation involving the nervous system being a hallmark of the condition. Treatments include medications such as disease-modifying antirheumatic drugs (DMARDs), corticosteroids, and biologics targeting inflammatory pathways. Yet, these treatments are not curative for RA. Heat Shock Proteins (HSPs) are molecular chaperones with immunoregulatory properties; however, their role is not yet fully understood, as these molecules may play a dual, pro- and anti-inflammatory role. In this study, we evaluated the protein expression levels of HSPs 27, 60, 70, and 90 in the synovial membrane and spinal cord of the RA rats’ model to determine their roles during the disease course, both on the neurological and immunological levels. Furthermore, HSP levels have been evaluated in the spinal cord of control and RA rats’ model after high and low doses of ketamine injection. Significant changes in Hsp60, 70, and 90 expression levels were observed only in the spinal cord of RA rats. We demonstrated that blocking N-methyl-D-aspartate receptors with ketamine can modulate spinal cord HSPs expression in RA rats and subsequently impact neurogenic inflammation and adult neurogenesis. This suggests that HSPs may be a promising target for RA treatment due to their complex immunomodulatory effects and potential interactions with the nervous system. Further research is needed to explore their therapeutic potential and develop effective interventions for RA.

## 1. Introduction

Rheumatoid arthritis (RA) is a chronic inflammatory autoimmune disease characterized by sustained joint inflammation. Approximately 3 in every 10,000 individuals are affected by RA annually around the world [1].

The disease progresses through four distinct stages: first, the immune system attacks joint tissues; then, the connective tissue swells and is damaged; third, an increased inflammation with distinctive physical deformities of cartilage is observed; lastly, the complete destruction of joints and cartilage occurs. RA is initiated by the immune system mistakenly targeting cells and proteins in the joint lining, resulting in a chronic autoimmune inflammatory state comprising different joint components [2,3]. Inflamed joint blood vessels become permeable, allowing invasion of activated T and B lymphocytes, macrophages, neutrophils, mast and dendritic cells, contributing to chronic autoimmune processes and a persistent inflammatory response [4]. These immune cells secrete pro-inflammatory cytokines and chemokines, further extending the inflammation within the synovial compartment [5,6,7]. Consequently, competing targeted therapeutics, such as anti-CD3, CTLA4-Ig, anti-TNF-α, or B–cell–depleting antibodies, have been used for the suppression of the inflammatory response [8]. Nevertheless, they do not alter immune tolerance.

As a result, several therapeutic approaches have shifted focus to finding novel ways to dampen this inflammation, restore immunotolerance, and re-establish physiological regulation. Hence, efforts have been made to target specific immunogenic proteins such as the heat shock proteins (HSPs) [9], a family of molecular chaperones that contribute to the immune responses as part of their non-canonical function. However, the interplay between HSPs and the immune system is intricate since the HSPs have been demonstrated to have a dual role, acting both as immunostimulatory and immunoregulatory factors [9]. While there is extensive literature on RA synovial membrane inflammation and HSPs [10], less attention has been directed towards their role in the central nervous system (CNS), particularly in the spinal cord during RA, where they may impact pain sensitization and neurological implications. RA’s development is influenced by neurogenic inflammation, a pathophysiological process involving complex responses from the immune, vascular, and neural systems [11,12,13]. In the context of RA, HSPs 27, 60, 70, and 90 have been described to exhibit immunoregulatory attributes. For example, Hsp70 proteins have been shown to downregulate inflammation in RA fibroblast-like synoviocytes. Nonetheless, these proteins also have a pro-inflammatory role, where they increase the frequency of Th17, thus enabling RA to progress [14]. Similarly, Hsp60 has been shown to empower the secretion of both the pro-inflammatory (IL-1β) and anti-inflammatory (IL-4 and IL-10) cytokines in RA [15,16]. On the other hand, N-methyl-D-aspartate receptor (NMDAR), a key glutamate receptor and ion channel, is also associated with altered expression of HSPs. For instance, neuroprotection against dementia-inducing neuroinflammatory proteins has been demonstrated by Hsp27 and Hsp70 through the activation of NMDAR in rat brain cultures [17,18]. The neuroinflammatory process increases afferent input induced by peripheral inflammatory mediators that affect pain processing and induce inflammation-related hyperalgesia [19,20], binding various CNS receptors, including NMDAR [21]. NMDAR plays a crucial role in spinal neurons’ heightened responses and basal activity induced by peripheral inflammation; indeed, glutamate receptor antagonists, specifically ones for NMDAR such as ketamine, inhibit the in vitro synovial fibroblast proliferation [22] and enhance the in vivo anti-arthritic activities of dexamethasone [23].

Moreover, a growing body of evidence has revealed the role of HSPs in inducing cellular proliferation under pathophysiological conditions. For example, the Hsp70 has been detected on the outer side of the neuroblast membrane, regulating cellular migration [17]. Administration of exogenous Hsp70 has been demonstrated to significantly increase the cognitive function in several mice, enabling novel object recognition and improving learning and memory consolidation capacities [18,24]. Furthermore, pharmacological induction of the Hsp70 gene is associated with cognitive improvement in brain-injured mice [25]. Thus, after assessing the effects of Complete Freund’s Adjuvants (CFA) injection on the rats’ joint circumference, motor coordination and nociceptive behavior, in this work we evaluated the effects of inflammation in the RA rats’ spinal cord, in terms of inflammation markers, astroglia cells expression, proliferating cells, and expression of HSPs 27, 60, 70, and 90. Although the CFA model does not fully replicate the autoimmune complexity of human rheumatoid arthritis, there is abundant evidence that CFA injection induces long—lasting pain hypersensitivity, glial activation, and metabolic and epigenetic changes lasting well into the chronic phase. Thus, CFA remains a valid model for studying disease initiation, progression, and neuroimmune modulation. Furthermore, low and high dose of ketamine on HSPs expression was explored in this study within the spinal cord, and the investigations were performed and compared to the rats not treated with ketamine. We validated that both low and high doses of ketamine might have distinct effects on behavior and spinal cord HSPs expression in RA rats, indicating potential in addressing pain sensitization as previously noted in chronic conditions [26]. Therefore, rather than directly targeting glutamate receptors, which could affect various CNS functions beyond pain modulation, focusing on HSPs and their regulation may offer a more refined therapeutic approach. It may allow for broader regulation of cellular stress responses and neuroprotective mechanisms, potentially leading to sustainable therapeutic outcomes in managing CNS manifestations of RA. Furthermore, we suggest that these changes are correlated, in a yet little-known way, with the development of nociceptive behavior and increased cellular proliferation within the spinal cord, as we reported here. This study does not negate the importance of HSPs in the synovial membrane, which has been extensively studied in the context of joint inflammation and local therapies [10]. Rather, it seeks to complement existing knowledge by exploring a less-explored aspect of RA pathophysiology, specifically focusing on the spinal cord as a site of potential HSP-mediated neuroprotective or pathological processes.

## 2. Results

### 2.1. The Effect of Complete Freund’s Adjuvants (CFA) Injection on Joint Inflammation

**Figure 1:**
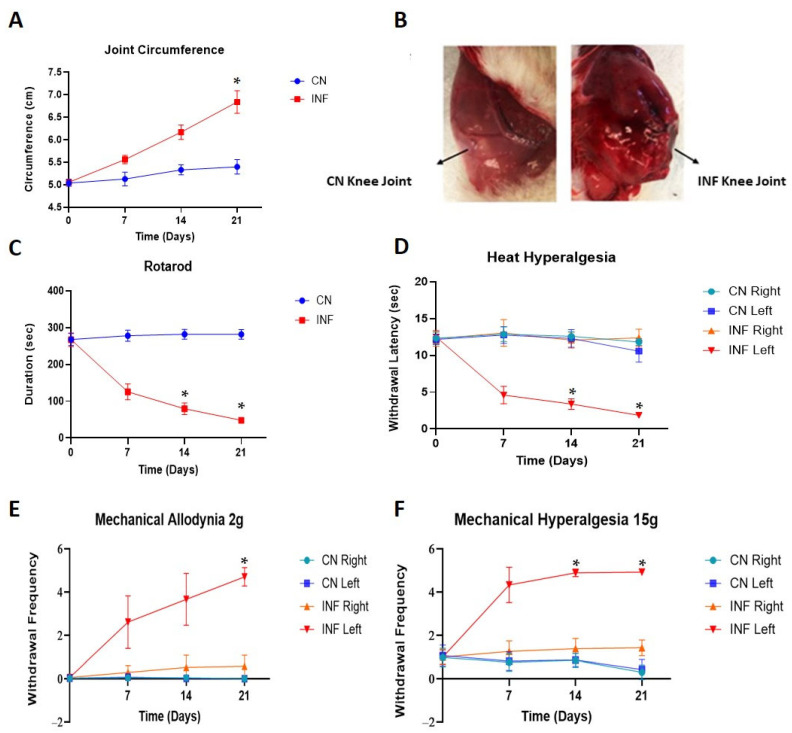
Effects of CFA injection on joint circumference, motor coordination, and nociceptive behavior. Line graphs representing results for CN (control) and INF (inflamed) groups, over time, n = 30. (**A**) Progression of the joint circumferences in CN and INF groups, over time. The joint circumferences are represented as the average circumference of the knee joint measured before the induction of inflammation and on days 7, 14, and 21 post-injection of either saline (control or CN group) or CFA (inflamed or INF group). Data are presented as Mean ± SD and were analyzed by one-way ANOVA with Post Hoc Bonferroni tests comparing joint circumferences at corresponding time points. A significant increase occurred for the arthritic rats compared with the controls (* *p* < 0.05). (**B**) The saline-injected joint of CN rat (left) compared to the CFA-injected joint of INF rat (right). An exacerbated edema is shown in the joint of INF rat. (**C**) A line graph showing the comparison of motor abilities of CN and INF groups of rats calculated as the time taken to lose their balance from rotarod. INF rats’ balance ability declined in time, and it is statistically significant on days 14 and 21 compared to CN rats (* *p* < 0.05). Data are expressed as mean ± SD. (**D**) The foot withdrawal latency to a radiant heat applied to the plantar surface of the hind paw was measured for both hind paws (left and right) of INF and CN rats. A statistically significant difference between INF posterior foot withdrawal latency compared to the other groups was measured (* *p* < 0.05). Data are expressed as mean ± SD. (**E**) The paw withdrawal frequency to a noxious stimulus was recorded for both hind paws (left and right) of CN and INF rats. The difference in the withdrawal frequency of INF left paw became significant on day 21 (* *p* < 0.05), compared to the other groups. Data are expressed as mean ± SD. (**F**) The paw withdrawal frequency to a non-noxious stimulus was recorded for both hind paws (left and right) of CN and INF groups. The paw withdrawal frequency of INF left was significantly raised at days 14 and 21 (* *p* < 0.05) compared to the other paws. Data are expressed as mean ± SD.

To evaluate the severity of the joint inflammation, joint circumferences of control (CN) and inflamed (INF) rats were assessed before the induction of inflammation and on days 7, 14, and 21 post-injections. The injection of 0.1 mL of CFA into the synovial cavity of the knee joint of rats produced a chronic inflammatory response characterized by swelling, plasma extravasation, and nociceptive behaviors. As shown in panel A of Figure 1, in the INF group, the joint circumference of the injected knee showed a remarkable linear increase in time, whereas the joint circumference of the left knee of the CN group did not increase over time. Moreover, on day 21, a significant difference (*p* < 0.05) can be seen between the joint circumference of the INF group (7 cm) and the CN group (5.5 cm). Furthermore, the joint of the CFA injection (INF group) showed an exacerbated edema when compared to the saline-injected joint (CN group) (Figure 1B).

### 2.2. The Effect of CFA-Induced Inflammation on Motor Coordination

A rotarod machine was used to assess the effect of chronic peripheral inflammation on motor ability. The CN rats maintained their motor abilities through the different time points, with the time taken to lose their balance remaining almost unchanged at 250 s (Figure 1C). The INF rats, instead, had a significant reduction in their motor ability, with a continuous decrease in maintaining the balance from 250 s at baseline (T0) to 110 and 50 s at day 14 and 21, respectively (Figure 1C). The decline in the duration of the INF rats to fall was statistically significant compared to the CN animals on both days 14 and 21, with *p* < 0.05.

### 2.3. Nociceptive Behavior Changes

#### 2.3.1. The Effect of CFA-Induced Inflammation on Heat Hyperalgesia

To evaluate the development of nociceptive behaviors following inflammation induction, the hind paws of rats were exposed to a painful light-based stimulus (heat hyperalgesia test, Figure 1D). The CFA injection into the knee joint significantly decreased (*p* < 0.05) the withdrawal latencies of the injected paw (INF left, Figure 1D) from 12 s at T0 to 1.5 s on day 21 (Figure 1D). However, no changes in the foot withdrawal latency were observed in the intact contralateral posterior paw (INF right, Figure 1D). In the CN group, both the saline-injected paw (CN left, Figure 1D) and the contralateral paw (CN right, Figure 1D) showed consistent withdrawal latencies throughout the different time points.

#### 2.3.2. The Effect of CFA-Induced Inflammation on Mechanical Allodynia and Mechanical Hyperalgesia

On days 7, 14, and 21 post-induction of inflammation, rats injected with CFA showed a remarkable increase in the withdrawal frequency of the injected paw (INF left, Figure 1E) when compared to the contralateral paw (INF right, Figure 1E). The difference in the withdrawal frequency became significant on day 21 (*p* < 0.05). In contrast, the saline-injected groups exhibited no change in the withdrawal frequency of either paw from T0 (CN right and CN left, Figure 1E). Similar results were seen for mechanical hyperalgesia (Figure 1F), where a significant rise in the paw withdrawal frequency was seen in the paws of the INF left group compared to the others, at days 14 and 21 (*p* < 0.05).

### 2.4. The Effect of CFA-Induced Inflammation on the Expression of HSPs in the Spinal Cord

**Figure 2:**
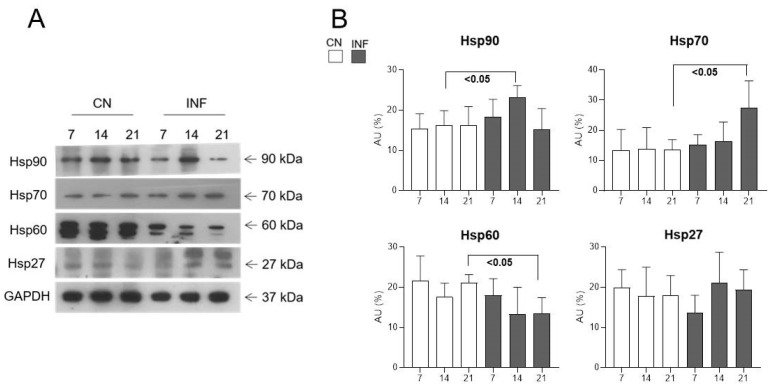
Protein expression levels of Hsp90, Hsp70, Hsp60, and Hsp27 in the spinal cord of CN and INF groups of rats. Rats injected in their left posterior knee joint with CFA are the inflamed group (INF) *n* = 15, those injected with saline are the control group (CN) *n* = 15. Representative Western Blot of Hsp90, Hsp70, Hsp60, and Hsp27 (arrows indicate proteins’ molecular weight) from the tissue of two groups is shown in (**A**) and relative expression levels are observed in (**B**). GAPDH was used as the loading control. Data are presented as the mean ± SD. Significantly different values are represented by *p* < 0.05.

The change in the expression levels of multiple HSPs centrally, in the spinal cord, and peripherally, in the synovial membrane, was assessed in the INF and CN rats at different time points. Western Blot analysis showed a significant increase in the expression of Hsp90 in the spinal cord of the INF rats at day 14 (*p* < 0.05) and of Hsp70 in the spinal cord of the INF rats at day 21 (*p* < 0.05), as seen in Figure 2, compared to the corresponding CN counterpart group. The Hsp60 levels showed a significant decrease on day 21 in the INF animals compared to the CN rats (*p* < 0.05), whereas Hsp27 protein levels remained unchanged at all time points (Figure 2). Baseline serves as a reference point against which experimental groups can be compared. Once the baseline measurements were established and confirmed, further experiments focused solely on the experimental group to explore the comprehensive insights into how RA influences HSP expression dynamics in the spinal cord, thereby contributing to the understanding of neuroinflammatory processes and potential therapeutic strategies.

**Figure 3:**
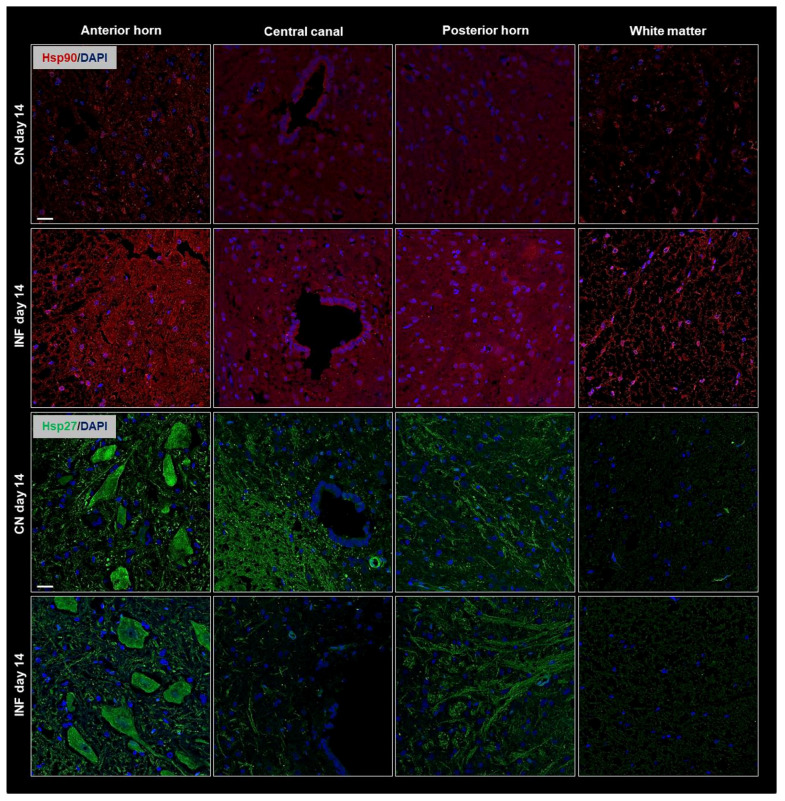
Immunofluorescence of Hsp90 and Hsp27 in the Anterior Horn, Central Canal, Posterior Horn, and White Matter of the spinal cord tissues from inflamed and control groups of rats. Rats injected in their left posterior knee joint with CFA are the inflamed group (INF) *n* = 15, and ones injected with saline are the control group (CN) *n* = 15. Immunofluorescence with TRITC-conjugated anti-Hsp90 (red) on sections at day 14 (**top panel**), and FITC-conjugated anti-Hsp27 antibody (green) on sections at day 14 (**bottom panel**) are shown; Nuclei have been stained with DAPI (blue signal). Scale bar = 25 µm.

**Figure 4:**
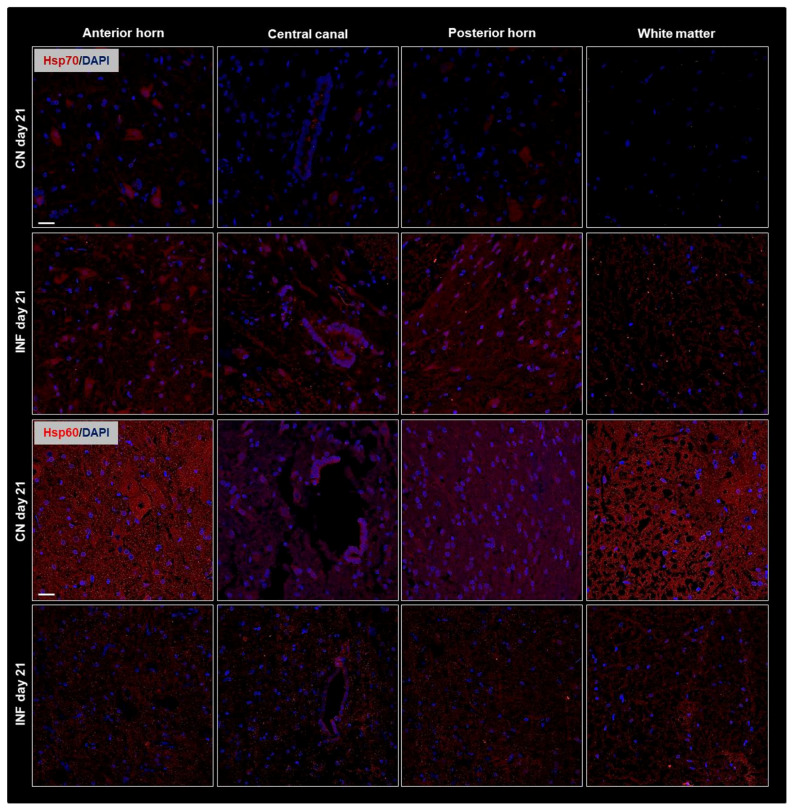
Immunofluorescence of Hsp70 and Hsp60 in the Anterior Horn, Central Canal, Posterior Horn, and White Matter of the spinal cord tissues from inflamed and control groups of rats. Rats injected in their left posterior knee joint with CFA are the inflamed group (INF), *n* = 15, and ones injected with saline are the control group (CN), *n* = 15. Immunofluorescence with TRITC-conjugated anti-Hsp70 (red) on sections at day 21 (**top panel**), and TRITC-conjugated anti-Hsp70 (red) on sections at day 21 (**bottom panel**) are shown; Nuclei were stained with DAPI (blue signal). Scale bar = 25 µm.

To complement the Western Blot findings and further validate our observations, immunofluorescence (IF) experiments were performed. Spatial resolution provided by IF experiments showed protein distribution in the different regions of the spinal cord, including the anterior horn, central canal, posterior horn, and white matter and further IF captured the dynamic changes in HSPs expression over time or in response to experimental conditions. The IF results consistently mirrored the patterns observed in the Western Blot analysis. Therefore, Results showed identical patterns to those of the Western Blot experiment with an increase in the signal levels of Hsp90 (Figure 3 top panel) and of Hsp70 (Figure 4 top panel) on days 14 and 21, respectively, in the different regions of the spinal cord of the inflamed rats compared to the sections from the spinal cord of the control group. Moreover, Hsp60 signal levels dropped after 3 weeks of inflammation induction (Figure 4 bottom panel) compared to saline-injected rats at 21 days, while those of Hsp27 remained invariable throughout the experimental timeline (Figure 3 bottom).

The expression of HSPs in synovial membranes extracted from the knee joint of both INF and CN groups was assessed to evaluate their peripheral abundance. Western Blot experiments showed no significant variation in the expression levels of the different HSPs at any of the studied time points (Figure S1).

### 2.5. The Effect of CFA-Induced Inflammation on the Expression of GFAP in the Spinal Cord

**Figure 5:**
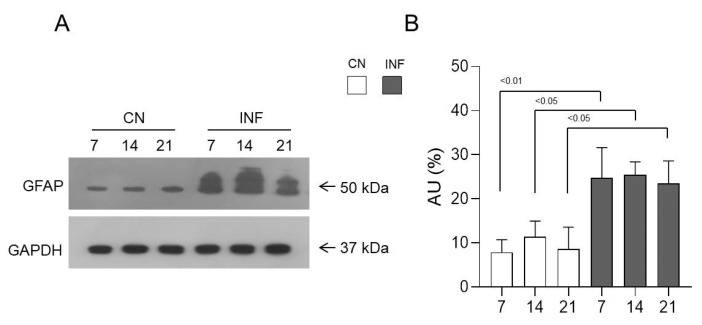
Protein expression levels of glial fibrillary acidic protein (GFAP) in the spinal cord of inflamed and control rats. GFAP expression level was measured in the spinal cord of the two groups of saline-injected rats (CN) *n* = 3, and CFA-injected (INF) *n* = 3. Representative Western Blot (arrows indicate proteins’ molecular weight) (**A**) and relative expression levels (**B**) of GFAP are shown for both groups. GAPDH was used as the loading control. Data are presented as the mean ± SD. Significantly different values are represented by *p* < 0.05 and *p* < 0.01.

To evaluate the expected changes in the levels of astroglia cells in the spinal cord tissues, Western Blot experiments were performed to detect their specific marker, known as the glial fibrillary acidic protein (GFAP). GFAP expression was significantly 3increased in the INF group compared to the CN group at each of the examined time points, with *p* < 0.01 at day 7 and *p* < 0.05 at days 14 and 21 (Figure 5). Thus, these cells are extensively present and activated in the inflamed spinal cord. This statement was consolidated by immunofluorescence data, which showed an increase in the expression of GFAP (Figure S2A–C of Supplementary Material).

The GFAP was observed at various time points (days 7, 14, and 21) in 3 distinct areas of the spinal cord: Posterior Horn, Central Canal, and Anterior Horn. To show that astroglia expressed the HSPs, double staining for GFAP and Hsp90, 70, and 60 was performed on spinal cord tissues, as shown in Figure S2A–C of the Supplementary Material. A detectable colocalization of GFAP and Hsp90, Hsp70, and Hsp60 in astroglia was detected, and a representative panel of their colocalization in the anterior horn, posterior horn, and central canal of the spinal cord from saline-injected rats at day 14 is shown in Figure S3A of the Supplementary Material.

Moreover, a thorough quantitative analysis of the immunofluorescence signals was performed on the two groups of animals to compare the colocalization of GFAP with the HSPs. Figure S3B of the Supplementary Material shows the colocalization rate of GFAP with HSPs (60, 70, and 90) in the three regions of the spinal cord, using Pearson’s correlation analysis and percentage of colocalization. No remarkable difference between saline and inflamed rats has been identified at each time point for any of the studied HSPs (Figure S3B of the Supplementary Material).

### 2.6. The Effect of CFA-Induced Inflammation on CD68-Immunopositivity in the Different Regions of the Spinal Cord

**Figure 6:**
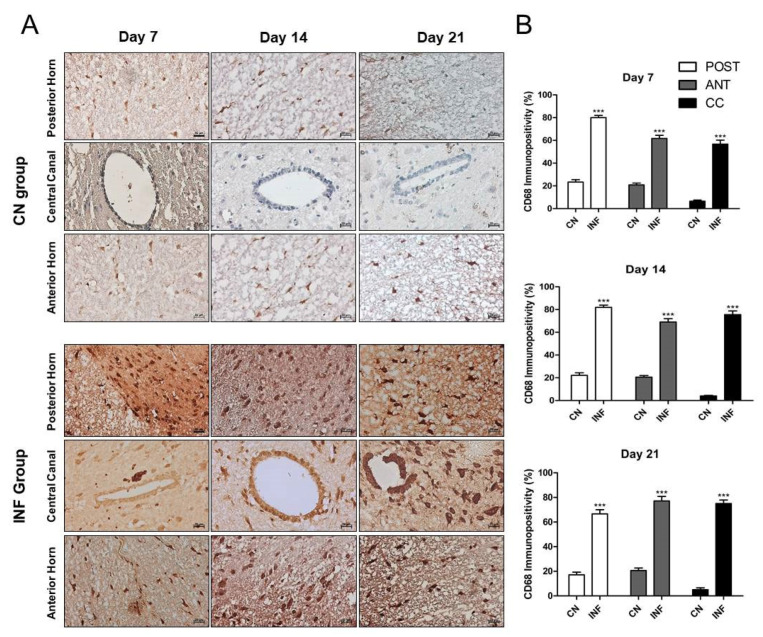
Immunohistochemistry of Cluster of Differentiation 68 (CD68) on spinal cord tissues from control and inflamed rats. Rats injected in their left posterior knee joint with CFA are the inflamed group (INF), *n* = 15, and ones injected with saline are the control group (CN), *n* = 15. The CD68 immunopositivity was detected in the anterior horn, central canal, and posterior horn of the spinal cord of CN and INF rats on days 7, 14, and 21 (**A**). CD68 immunopositivity percentage analyzed on days 7, 14, and 21 in the Posterior Horn (Post), Anterior Horn (Ant), and Central Canal (CC) from the spinal cord of the CN and INF groups of rats is shown (**B**). Magnification 400X, scale bar 20 µm. Data are presented as the mean ± SD. *** *p* < 0.001.

CD68-expressing microglia/macrophages are considered one of the crucial markers in animals with chronic inflammation. Therefore, the expression level of CD68 was measured in this study. Immunohistochemistry results showed a significant increase in CD68 immunopositivity in the Anterior Horn, Posterior Horn, and Central Canal in the animals injected with CFA (INF) when compared to those injected with saline (CN) on days 7, 14, and 21 (Figure 6A). This increase was statistically significant (*p* < 0.001) in all different regions and throughout the experimental timeline (Figure 6B).

### 2.7. The Effect of CFA-Induced Inflammation on Cellular Proliferation Detected by BrdU-Positive Cells in the Spinal Cord

**Figure 7:**
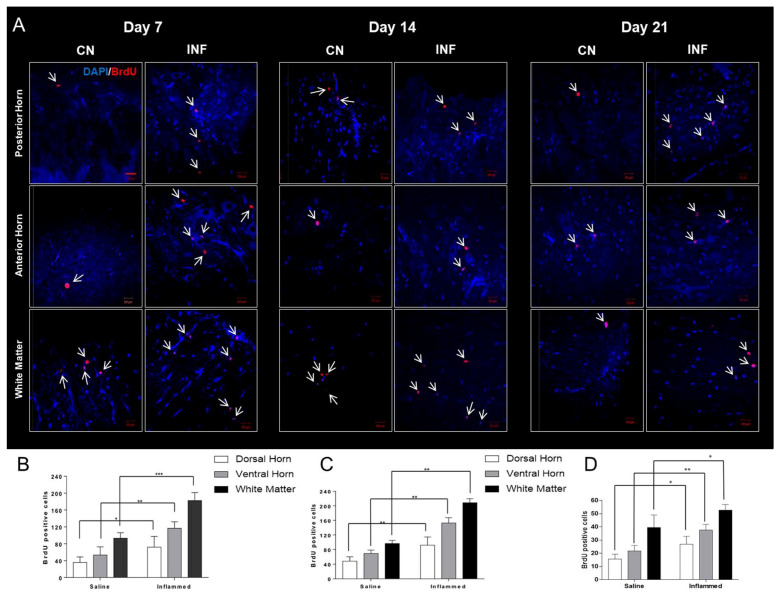
BrdU-positive cells in different regions of the lumbar spinal cord in inflamed and saline-injected rats on days 7, 14, and 21 post-injection. (**A**) Representative panels showing BrdU-positive nuclei (red) indicated by the white arrows, and DAPI (blue) cells in the posterior horn, anterior horn, and white matter of the spinal cord from saline-treated (control, CN) (*n* = 9) and CFA-treated (inflamed, INF) rats (*n* = 9) on days 7, 14, and 21. (**B**) quantitative comparisons of the total number of BrdU-positive cells in saline versus inflamed rats in the aforementioned regions of the spinal cord at day 7. (**C**) quantitative comparisons of the total number of BrdU-positive cells in saline versus inflamed rats in the aforementioned regions of the spinal cord at day 14. (**D**) quantitative comparisons of the total number of BrdU-positive cells in saline versus inflamed rats in the aforementioned regions of the spinal cord at day 21. Data were expressed as means + SEM. White arrows indicate BrdU-positive cells. * *p* < 0.05; ** *p* < 0.01; *** *p* < 0.001. Scale bar, 20 μm.

5-bromo-2-deoxyuridine (BrdU) injections were carried out to visualize the distribution of proliferating cells in various parts of the spinal cord. BrdU-positive cells were counted using confocal microscopy. At the different time points, an increase in cellular proliferation was observed in inflamed rats compared to saline-treated animals in the different regions of the spinal cord: the dorsal horn, ventral horn, and white matter (Figure 7). The number of proliferating cells in the L3–L4 segment located in the middle of the lumbar spine was quantified. As seen in Figure 7A, BrdU-positive cells were significantly higher on days 7, 14, and 21 in the CFA-injected group (INF) as compared to the saline-injected (CN) group. On days 7, 14, and 21, the upsurge in cellular proliferation was significantly enhanced in the posterior horn, anterior horn, and white matter (*p* < 0.05) (Figure 7B–D). Additionally, BrdU-labeled cells were occasionally noted in the ependymal layer of the Central Canal.

### 2.8. The Effect of the NMDA Receptor Antagonist Ketamine on the Expression of HSPs in the Spinal Cord

#### 2.8.1. Motor Behavioral Testing

**Figure 8:**
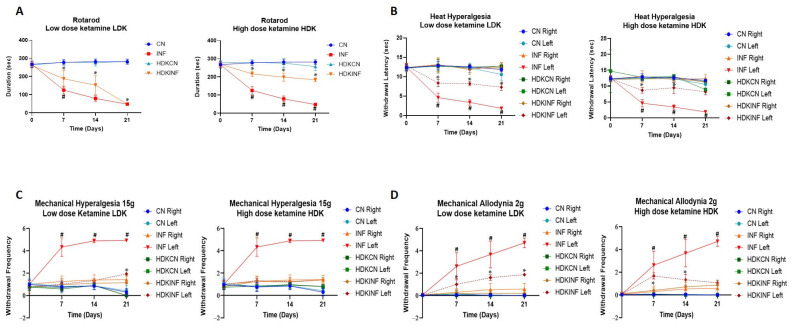
Time course of the effect of chronic peripheral inflammation on motor coordination in rats injected with high and low doses of ketamine. Rats injected with a low (*n* = 9) or high (*n* = 9) dose of ketamine and with CFA in the posterior left knee synovial cavity are the inflamed group (LDKINF and HDKINF, respectively), while rats injected with a low *(n* = 9) or high dose (*n* = 9) of ketamine and with saline in the posterior left knee synovial cavity are the control group (LDKCN and HDKCN, respectively). (**A**) A line graph showing the comparison of motor abilities of the CN, INF, LDKCN, HDKCN, LDKINF, and HDKINF group of rats, calculated as the time taken to lose their balance from rotarod. Data are expressed as mean ± SD. * LDKINF vs. LDKCN and HDKINF vs. HDKCN *p* < 0.01; # LDKINF vs. INF and HDKINF vs. INF *p* < 0.01. (**B**) The foot withdrawal latency to a radiant heat applied to the plantar surface of the hind paw was measured for both hind paws (left and right) of CN, INF, LDKCN, HDKCN, LDKINF, and HDKINF groups rats. Data are expressed as mean ± SD. * LDKINF left vs. LDKCN left and HDKINF left vs. HDKCN left *p* < 0.01; # INF left vs. LDKINF left, and INF left vs. HDKINF left *p* < 0.005. (**C**) The paw withdrawal frequency to a noxious stimulus was recorded for both hind paws (left and right) of the CN, INF, LDKCN, HDKCN, LDKINF, and HDKINF rats. Data are expressed as mean ± SD. * LDKCN left vs. LDKINF left *p* < 0.005; # INF left vs. LDKINF left and INF left vs. HDKINF left *p* < 0.0001. (**D**) The paw withdrawal frequency to a non-noxious stimulus was recorded for both hind paws (left and right) of the CN, INF, LDKCN, HDKCN, LDKINF, and HDKINF groups. Data are expressed as mean ± SD. * LDKCN left vs. LDKINF left and HDKCN left vs. HDKINF left *p* < 0.005.

The rotarod test is one of the most used techniques in assessing the effects of a drug on animal motor behavior [27]. INF rats showed a significant decrease in motor ability on day 7 compared to LDKINF (*p* < 0.01), as seen in Figure 8A. While INF rats showed a significant decrease in motor ability on days 7, 14, and 21 when compared to HDKINF (*p* < 0.01) (Figure 8A). LDKINF and HDKINF rats showed a decrease in motor ability on days 7, 14, and 21 compared to LDKCN and HDKCN rats, respectively (*p* < 0.01) (Figure 8A).

#### 2.8.2. Heat Hyperalgesia

As observed in the left panel of Figure 8B, intraperitoneal injection of low-dose ketamine in rats showed a significant decrease in withdrawal latency in LDKINF left compared to LDKCN left on days 7, 14, and 21 (*p* < 0.01). Similar results are shown in HDKINF left compared to HDKCN left only on days 7 and 14 (Figure 8B). Both left paws of LDKINF and HDKINF rats showed a decrease in withdrawal latency on days 7, 14, and 21 compared to INF left rats’ paws (*p* < 0.005) (Figure 8B).

#### 2.8.3. Mechanical Hyperalgesia

On days 7, 14, and 21, INF left rats’ paw withdrawal frequency increased compared to LDKINF left and HDKINF left groups (*p* < 0.0001) (Figure 8C). Results also revealed a significant increase in withdrawal frequency in the LDKINF (left hind) compared to LDKCN left (Figure 8C left panel) (*p* < 0.005).

#### 2.8.4. Mechanical Allodynia

On days 7, 14, and 21, INF left rats’ paws withdrawal frequency increased compared to LDKINF left and HDKINF left groups (*p* < 0.0001) (Figure 8D). On the other hand, LDKINF and HDKINF left paw withdrawal frequency increased compared to LDKCN and HDKCN (left paw), respectively, on days 7 and 14 (*p* < 0.005), while LDKINF left paw withdrawal frequency significantly increased compared to LDKCN (left paw) on day 21 (*p* < 0.005) (Figure 8D).

#### 2.8.5. Expression of HSPs in the Spinal Cord of Control and Inflamed Rats Treated with Low and High Doses of Ketamine

**Figure 9:**
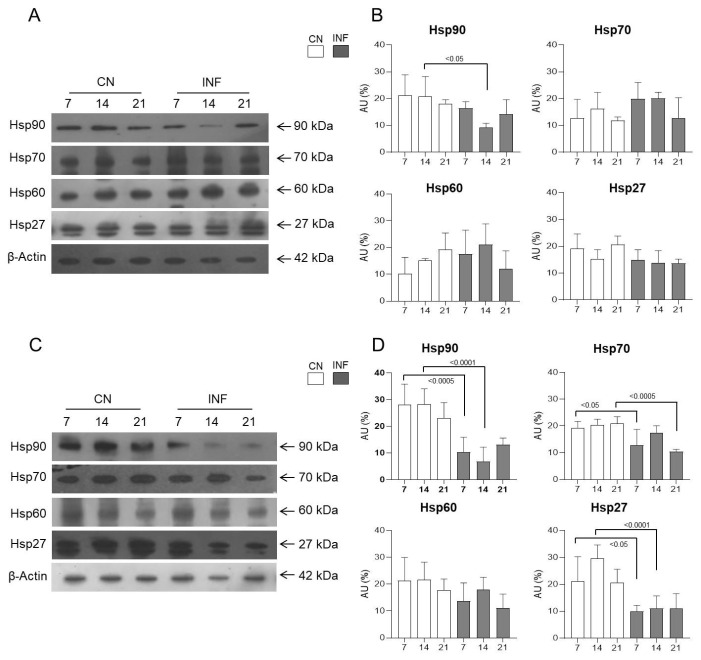
Protein expression levels of Hsp90, Hsp70, Hsp60, and Hsp27 in the spinal cord of control and inflamed rats treated with low and high doses of ketamine. Rats injected in their left posterior knee joint with CFA and with a low (*n* = 9) or high (*n* = 9) dose of ketamine are the inflamed group (LDKINF and HDKINF, respectively); rats injected with saline and with a low (*n* = 9) or high (*n* = 9) dose of ketamine are the control group (LDKCN and HDKCN, respectively). (**A**,**B**) show the result of Western blot and relative protein expression levels of Hsp90, Hsp70, Hsp60, and Hsp27 from the spinal cord of LDKCN and LDKINF rats. Arrows indicate the molecular weight of the investigated protein. (**C**,**D**) show the result of Western blot and relative protein expression levels of Hsp90, Hsp70, Hsp60, and Hsp27 from the spinal cord of HDKCN and HDKINF rats. β-Actin was used as a loading control. Data are presented as the mean ± SD. Statistically different values are represented by *p* < 0.05, *p* < 0.0005, and *p* < 0.0001.

In rats injected with saline and CFA, and with a low dose of ketamine, therefore belonging to LDKCN and LDKINF, Western blot experiments from spinal cord protein extraction showed no significant variation in the expression of HSPs 27, 60, and 70 (Figure 9A,B). While Hsp90′s expression level significantly decreased in LDKINF rats compared to LDKCN rats at day 14 (*p* < 0.05), as seen in Figure 9A,B. However, animals receiving saline and CFA, and high doses of ketamine showed significant variations in the expression of HSPs 90, 70, and 27 in the HDKINF rats compared to the HDKCN rats (Figure 9C). For Hsp90, a significant decline was seen in the HDKINF rats on days 7 and 14 with *p* < 0.0005 and *p* < 0.0001, respectively (Figure 9D). A similar pattern was seen for Hsp27, with significant decreases on day 7 (*p* < 0.05) and on day 14 (*p* < 0.0001) as seen in Figure 9D. A significant decrease in the expression of Hsp70 occurred on day 7 (*p* < 0.05) and day 21 (*p* < 0.0005) in the HDKINF animals (Figure 9D).

#### 2.8.6. CD68-Immunopositivity in the Different Regions of the Spinal Cord of Rats Treated with Ketamine

**Figure 10:**
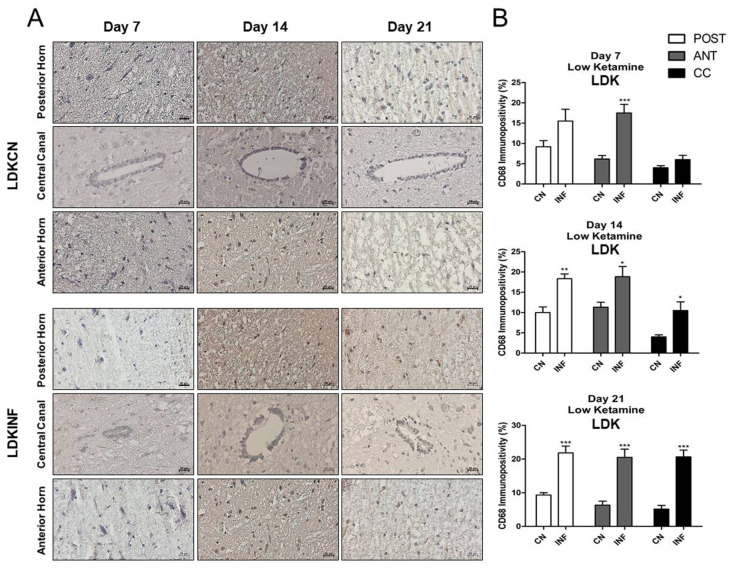
Immunohistochemistry of Cluster of Differentiation 68 (CD68) on Spinal Cord sections in control and inflamed rats treated with low-dose ketamine. Rats injected in their left posterior knee joint with CFA and with a low dose of ketamine are the inflamed group (LDKINF) (*n* = 9); rats injected with saline and with a low or high dose of ketamine are the control group (LDKCN) (*n* = 9). The CD68 immunopositivity was detected in the anterior horn (ANT), central canal (CC), and posterior horn (POST) of the spinal cord of LDKCN and LDKINF rats on days 7, 14, and 21 (**A**). CD68 immunopositivity percentage analyzed on days 7, 14, and 21 in the posterior horn (POST), anterior horn (ANT), and central canal (CC) from the spinal cord of the LDKCN and LDKINF of rats is shown (**B**). Magnification 400X, scale bar 20 µm. Data are presented as the mean ± SD. * = *p* < 0.05; ** *p* < 0.01; *** *p* < 0.001.

**Figure 11:**
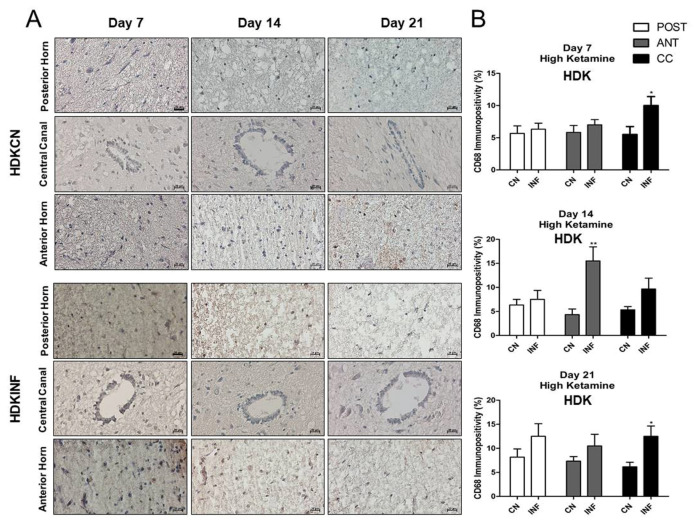
Immunohistochemistry of Cluster of Differentiation 68 (CD68) on Spinal Cord sections in control and inflamed rats treated with high-dose ketamine. Rats injected in their left posterior knee joint with CFA and with a high dose of ketamine are the inflamed group (HDKINF) (*n* = 9); rats injected with saline and with a low or high dose of ketamine are the control group (HDKCN) (*n* = 9). The CD68 immunopositivity was detected in the anterior horn (ANT), central canal (CC), and posterior horn (POST) of the spinal cord of HDKCN and HDKINF rats on days 7,14, and 21 (**A**). CD68 immunopositivity percentage analyzed on days 7,14, and 21 in the posterior horn (POST), anterior horn (ANT), and central canal (CC) from the spinal cord of the HDKCN and INF groups of rats is shown (**B**). Magnification 400X, scale bar 20 µm. Data are presented as the mean ± SD. * = *p* < 0.05; ** *p* < 0.01.

Qualitative and quantitative analyses showed that the injection of high-dose ketamine was able to lessen the impact of inflammation in the spinal cord. The level of CD-68-positive cells increased significantly on day 7 (*p* < 0.001), day 14 (*p* < 0.05), and day 21 (*p* < 0.001) in the anterior horn of LDKINF rats. Moreover, in LDKINF rats, the level of CD-68 immunopositivity increased at the level of the posterior horn at day 14 (*p* < 0.01) and day 21 (*p* < 0.001) and in the central canal at day 14 (*p* < 0.05) and day 21 (*p* < 0.001) (Figure 10A,B). A high dose of ketamine, however, decreased the presence of CD68-positive cells in the spinal cord in the HDKINF animals and brought their percentages down to values comparable to those found in the HDKCN animals (Figure 11A,B). Exceptions were found in the central canal on days 7 and 21 (*p* < 0.05) and the anterior horn on day 14 (*p* < 0.01) (Figure 11A,B).

#### 2.8.7. Comparisons of CD68-Immunopositivity in the Different Regions of the Spinal Cord of All Animal Groups

**Figure 12:**
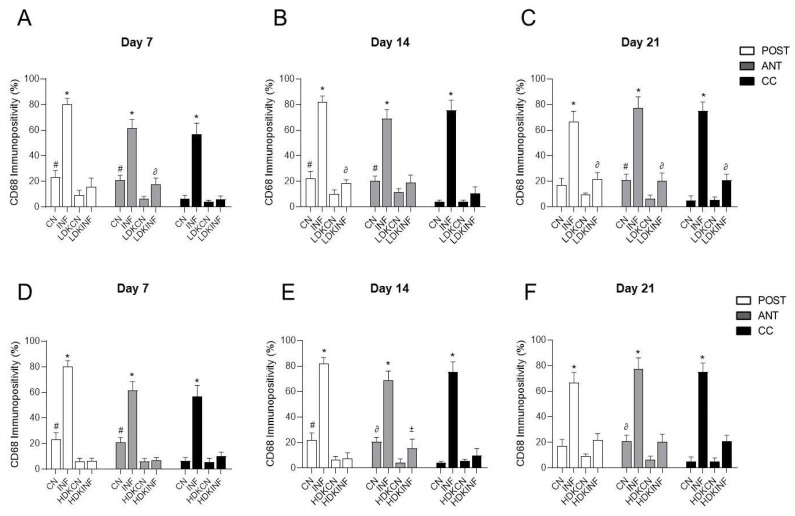
Immunopositivity of Cluster of Differentiation 68 (CD68) on the Spinal Cord section in control and inflamed rats treated with low and high-dose ketamine. Rats injected in their left posterior knee joint with CFA and with a low or high dose of ketamine are the inflamed groups (LDKINF) (HDKINF) (*n* = 9 each group); rats injected with saline and with a low or high dose of ketamine are the control groups (LDKCN) (HDKCN) (*n* = 9 each group). The CD68 immunopositivity was detected in the anterior horn (ANT), central canal (CC), and posterior horn (POST) of the spinal cord of the different groups on days 7, 14, and 21 ((**A**) of Figure 7, Figure 11, and Figure 12). CD68 immunopositivity percentages analyzed on days 7, 14, and 21 in the posterior horn, anterior horn, and central canal from the spinal cord of the CN, INF, LDKCN, LDKINF, HDKCN, and HDKINF of rats are shown (**A**–**F**). Magnification 400X, scale bar 20 µm. Data are presented as the mean ± SD. * *p* < 0.0001 (INF vs. CN, LDKCN, LDKINF) (INF vs. CN, HDKCN, HDKINF); # *p* < 0.0001 (CN vs. LDKCN, LDKINF) (CN vs. HDKCN, HDKINF); ∂ *p* < 0.005 (LDKINF vs. LDKCN); ∂ *p* < 0.0005 (CN vs. HDKCN); ± *p* < 0.01 (HDKINF vs. HDKCN).

To further validate ketamine’s anti-inflammatory role, quantitative analysis of CD68-immunopositive cells was performed across all groups. In the low-dose ketamine study, CD68 immunopositivity was significantly elevated in the inflammation-only (INF) group compared to control (CN), low-dose ketamine control (LDKCN), and low-dose ketamine with inflammation (LDKINF) groups across all spinal cord regions—posterior horn, anterior horn, and central canal—on days 7, 14, and 21 (*p* < 0.0001). However, the LDKINF still exhibited significantly higher CD68 immunopositivity than the LDKCN at specific time points: in the anterior horn on day 7, the posterior horn on day 14, and in all three regions on day 21 (*p* < 0.005), indicating a partial persistence of inflammation despite low-dose ketamine treatment. (Figure 12A–C).

A similar trend was observed in the high-dose groups. The INF group consistently showed significantly higher CD68 immunopositivity than CN, high-dose ketamine control (HDKCN), and high-dose ketamine with inflammation (HDKINF) groups across all spinal cord regions and time points (*p* < 0.0001). High-dose ketamine alone (HDKCN) significantly reduced CD68 immunopositivity in the posterior and anterior horns on day 7, and in the posterior horn on day 14, compared to CN rats (*p* < 0.0001). On day 14, HDKINF rats displayed significantly higher CD68 immunopositivity in the anterior horn compared to HDKCN (*p* < 0.01), again reflecting partial persistence of inflammation despite high-dose ketamine treatment. (Figure 12D–F).

## 3. Discussion

A plethora of studies investigating the mechanisms underlying the development and progression of RA have emphasized the vital role of specific members of the HSP family [9,10,13,14,15,16].

Nevertheless, the role of HSPs is still inconclusive. They might be directly involved in pathogenesis or have an indirect modulatory function. Moreover, they might be altered through specific immunoregulatory pathways and receptors [28]. In the present study, a rat model of RA was used to investigate the changes in the expression of different HSPs in this disease, both peripherally and centrally, as well as the motor coordination, nociceptive behavior, and tissue inflammation linked to these alterations. Moreover, we studied the expression of HSPs in the spinal cord and repeated the motor coordination and nociceptive behavior after injection of low or high doses of ketamine.

The CFA model was chosen to study the implications of HSPs in RA, as it is reported to show peripheral inflammation and activation of the immune cells with an increased nociceptive sensitivity [29]. The model was validated by a significantly increased joint circumference in the CFA-injected rats, accompanied by a chronic inflammatory response characterized by swelling, plasma extravasation, and nociceptive behaviors, as reported in the literature [30]. Enhanced nociceptive sensitivity in this model was proven through behavioral tests. The CFA-injected animals showed significantly decreased latencies of the injected paw withdrawal compared to the baseline values assessed from the contralateral paw, and from both paws of the control during the heat hyperalgesia experiment. Moreover, post-induction of inflammation, the injected paw of the inflamed rats showed significantly increased frequencies of withdrawal in both the mechanical allodynia and mechanical hyperalgesia tests compared to the control groups. Finally, motor coordination was lessened in the inflamed rats compared to their control counterparts as seen via the rotarod test.

As HSPs possess specific dual roles in the inflammation process, our study aimed to evaluate how various HSPs are particularly involved in RA pathogenesis and its subsequent neurogenic inflammation. While other studies have primarily focused on HSP expression in peripheral tissues such as the synovial membrane [31,32], in our work, the protein levels of HSPs 27, 60, 70, and 90 showed no variance in the synovial membrane of either control or inflamed rats (Figure S1) suggesting that joint inflammation in this model, and at this disease stage, may be mediated by factors other than HSPs. In the present study, we focused on the spinal cord as part of the central nervous system to investigate the role of HSPs in neuroimmune modulation, highlighting central mechanisms in rheumatoid arthritis. Our results showed that all the investigated HSPs, except Hsp27, showed fluctuating expression levels in the spinal cord. Western blot experiments showed an increased expression of Hsp90 and Hsp70 in inflamed rats on days 14 and 21, respectively, while Hsp60 levels showed a decrease in expression at day 21 in arthritic animals. When correlating the change in protein levels of these proteins with the timeline of the disease, it can be deduced that Hsp90 and Hsp70 may play pro-inflammatory and disease-enhancing roles as they rise at the onset of the disease. At the same time, the decreased expression of Hsp60 at day 21 (peak of inflammation) may play an anti-inflammatory role (Figure 2). These data were confirmed by IF detections of the HSPs in the anterior horn, central canal, posterior horn, and white matter of inflamed rats (Figure 3 and Figure 4). Taken together with findings from other studies [33,34], the potential modulatory role of the HSPs in regulating inflammatory responses in rheumatoid arthritis appears significant and warrants further in-depth investigation to better understand their contribution to disease mechanisms and therapeutic potential.

Since the chief component of RA is chronic inflammation, innate immune cells such as glial cells have a critical role in the persistence of the disease phenotype [35]. Glia also contributes to the development of damage signaling responses involved in tissue degeneration and nociceptive plasticity along the pain pathway [35]. Furthermore, seminal studies show that under conditions of acute cellular stress, neurons are inherently poor activators of the heat shock response, whereas astroglia readily activate it [36]. Thus, the colocalization of heat shock protein with astroglial cells was studied. Data showed a significant increase in GFAP expression level in the inflamed group versus saline at the three different time points (Figure 5), along with a trend toward increased colocalization rate with the different heat shock proteins (Figure S2A–C of Supplementary Material). Therefore, astroglia might be contributing to the pathogenesis of RA through their improved expression of HSPs in the spinal cord. On the other hand, macrophages and microglia in the CNS play key roles in pain initiation and its maintenance [35]. They are typically involved at the level of the dorsal root ganglion, where they aid in the induction and maintenance of the mechanical hypersensitivity that develops after chemotherapy models, nerve injury, and joint pathology [37]. Thus, CD68-expressing microglia/macrophages were investigated in this study. Our results showed a vast increase in CD68-expressing microglia/macrophages in the spinal cords of the rats injected with CFA compared with the group injected with saline (Figure 6). Hence, these cells might be a powerful tool that provokes RA symptoms. Altogether, these data suggest that CD68 microglia/macrophages seem to infiltrate the inflamed spinal cord and HSPs appear to be increased, as GFAP/HSPs colocalization in the inflamed spinal cord; however, the more elevated expression of certain HSPs and GFAP-positive cells may only contribute to the general increase in HSP observed in the Western blot assay and other CNS cells, yet not investigated here, could assist the rise in HSPs expression promoting the neurogenic inflammation.

Increased cellular proliferation in the different regions of the spinal cord post-induction of inflammation was studied in different models of arthritis. Its vital role in memory and learning systems, neuroplasticity, as well as in protecting the central nervous system from stress-induced attrition has been addressed [38]. In RA, this transient receptive field enhancement might acquire a consolidated state, as suggested by preclinical studies, where the ongoing inflammatory process in the affected limb promotes nerve damage and axonal sprouting, leading to permanent neuroplasticity, which characterizes the chronic pain phenotype [39]. In this study, the increase was correlated with the severity of the inflammation and the altered expression of HSPs. The BrdU, a thymidine analog, is a synthetic nucleoside widely employed for detecting proliferating cells within living tissues [40]. Upon intraperitoneal injection, BrdU becomes incorporated into the newly synthesized DNA of replicating cells, effectively substituting for thymidine during the process of DNA replication. The number of BrdU-expressing cells was high in the different regions of the spinal cord (dorsal horn, ventral horn, and the white matter) in inflamed rats versus saline in the different regions of the spinal cord at all time points (Figure 7). Our findings suggest that the increased proliferation under inflammatory conditions is correlated with the altered expression of specific HSPs that are considered neuromodulators and could contribute to the reorganization of dorsal horn cells, causing plastic changes that enhance nociceptive sensitivity.

Glutamate is known to be the main excitatory neurotransmitter in the mammalian CNS, playing key roles in memory, neuronal development, and synaptic plasticity [41]. Excessive glutamate release has been implicated in neuronal cell death. The NMDAR is one of the three types of ionotropic glutamate receptors, alongside the AMPA and kainate receptors. NMDARs are well-known to be involved in pain associated with peripheral tissue injury or nerve injury [42]. Permanent activation of NMDARs causes the spinal cord neurons to become more responsive to inputs, leading to the central sensitization component of chronic pain, characteristic of RA [43]. The link between NMDARs and HSPs in the CNS has been studied throughout the years. As stated by A. Tamura, high-affinity NMDAR receptor antagonists like MK-801 are known to induce Hsp70 in the posterior cingulate cortex and retrosplenial cortex of the rat brain [44]. NMDAR effects on the peripheral knee joints have also been studied by direct injection of low-dose ketamine into the synovial membrane in an Osteoarthritis (OA) model, leading to a decreased intra-articular pain in the sodium monoiodoacetate OA model [45]. Another study showed that intra-articular injection of ketamine ameliorated the pathological characteristics of OA, decreased TNF-α and NF-κB p65 expression levels, and increased the level of IL-10 expression in a dose-dependent manner [46]. In this study, to investigate the involvement of glutamate receptors in mediating the alteration of HSPs in both CFA-treated and saline-treated rats, low and high doses of ketamine injections were administered. Ketamine, known for its analgesic and anesthetic properties, also possesses the ability to inhibit NMDAR. After injection of high and low doses of ketamine in inflamed rats, behavioral changes occurred, providing the rats the ability to maintain their balance on the rotarod for a longer time compared to inflamed non-ketamine-injected rats, even if their resistance was lower than the control ketamine-injected rats. Moreover, the alterations were specifically noticeable in heat hyperalgesia and mechanical hyperalgesia tests, where, overall, the withdrawal latency to a radiant heat and withdrawal frequency to a noxious stimulus of the left CFA-injected paw was significantly altered in inflamed rats injected with a low and high dose of ketamine compared to inflamed not-ketamine-injected rats. Therefore, these changes suggest that the blocking of NMDARs may be beneficial for RA rats’ motor ability (Figure 8). In addition, a noteworthy change in the expression of heat shock proteins was also evaluated in ketamine-treated rats. Low-dose ketamine injections in rats showed a significant decrease in Hsp90 levels on day 14 in inflamed rats compared to control rats, while no variations of HSPs 27, 60, and 70 were detected when low-dose ketamine was injected. When high doses of ketamine were injected, inflamed rats showed a significant decrease in HSPs 90 and 27 on both days 7 and 14. Hsp70 showed a significant decrease on days 7 and 21. While Hsp60 showed no significant changes (Figure 9). These results suggest that NMDARs play an essential role in inducing the expression of HSPs in the CNS under pathophysiological conditions. We demonstrated that ketamine may induce a decrease in pro-inflammatory HSPs such as HSPs 90 and 70. Together, these findings suggest that ketamine not only acts through neuronal mechanisms but may also shift the balance of HSP function. Hsp90 and Hsp70 are often linked to pro-inflammatory signaling through their role in stabilizing IKK/NF-κB complexes and promoting cytokine release [47], while Hsp60 and other chaperones have been reported to exert anti-inflammatory and cytoprotective effects. By reducing TNF-α and IL-6 while enhancing IL-10 [48], ketamine may indirectly alter the “set point” of HSP expression and function in favor of an anti-inflammatory phenotype. Beyond cytokine regulation, ketamine also exerts effects on neuronal excitability at the spinal level [49]. It is well established that inflammatory mediators enhance neuronal activity, which in turn aggravates peripheral inflammation through dorsal root reflexes and neuropeptide release. By dampening excitability via NMDA receptor antagonism, ketamine may act on a common pathway underlying both inflammatory pain and the maintenance of inflammation. This aligns with evidence from preclinical models showing that NMDA receptor blockade reduces central sensitization and inflammatory joint damage [46,50]. Thus, ketamine’s dual action—limiting central sensitization and altering cytokine/HSP networks—positions it as a promising modulator of neuroimmune crosstalk. In light of this, we are aware of the limitations of this study section, that is, the absence of a direct comparison between the CN and INF groups, not treated with ketamine, and the ketamine-treated groups (LDKCN, LDKINF, HDKCN, and HDKINF), which would further be addressed in future studies. Yet using this dose-dependent approach allowed us to understand a broader spectrum of ketamine’s effects and helped identify potential threshold levels for therapeutic efficacy in the rheumatoid arthritis model. Finally, we showed that low and high doses of ketamine can counteract the effect of the CFA, inducing a decrease in CD-68 immunopositivity in the spinal cord sections of RA rats, in all examined time points. These results suggest that NMDARs might be partaking in the process of macrophage/microglia activation through HSP enrichment, as all rats receiving ketamine injections showed a decrease in the expression of CD68-expressing microglia/macrophages in the CNS (Figure 10, Figure 11 and Figure 12).

## 4. Materials and Methods

### 4.1. Animal Model

**Figure 13:**
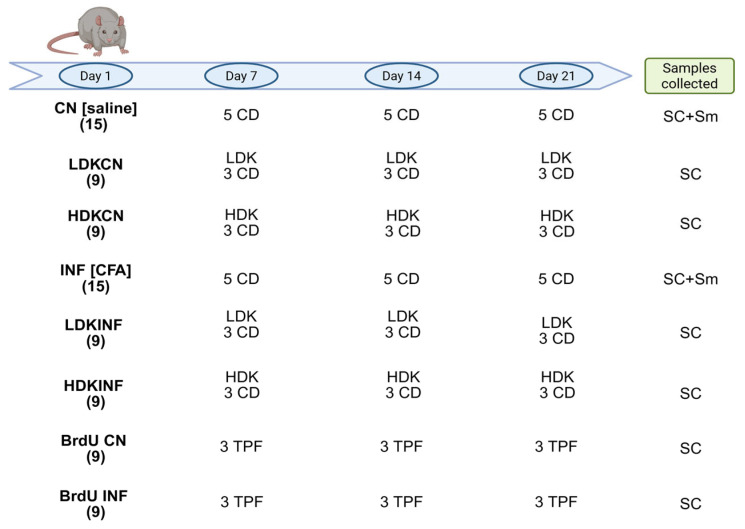
Experimental design. The procedures carried out on days 1, 7, 14, and 21 and the samples collected after rats’ euthanasia are reported. In brackets, in bold, the number of rats per group. CN, control, rats treated with saline solution; LDKCN, low dose ketamine and saline solution; HDKCN, high dose ketamine and saline solution; INF, inflamed, rats treated with Complete Freund’s Adjuvants (CFA); LDKINF, low dose ketamine and CFA; HDKINF, high dose ketamine and CFA; BrdU, 5-bromo-2′-deoxyuridine; CD, cervical dislocation; TPF, transcardial perfusion; SC, spinal cord; Sm, synovial membrane. Created in BioRender. Caruso Bavisotto, C. (2025) https://BioRender.com/hk38vr5 (accessed on 8 July 2025).

Eight-week-old Sprague Dawley male rats were used in this study. Rats were divided randomly into two groups (Figure 13): the control (CN) group (*n* = 15), rats were injected at day 1 with saline; the inflamed (INF) group (*n* = 15), rats were injected at day 1 with the inflammatory agent Complete Freund’s Adjuvants (CFA) (AdjuLite™ Complete Freund’s Adjuvant; Pacific Immunology Corp., Ramona, CA, USA). Other 36 rats were injected with saline and CFA after ketamine ((Ketamine; 50 mg/kg, Panpharma, Luitré, France) injections (groups LDKCN, HDKCN, LDKINF, HDKINF), as described below in Section 4.2. CFA is a suspension of desiccated mycobacterium in paraffin oil and mannide monooleate that induces inflammation, tissue necrosis, and ulceration [51]. A 1 mL syringe was used to inject 0.1 mL of CFA into the inflamed group’s posterior left knee synovial cavity. Control rats received an equivalent volume of saline at the same injection site. All rats were euthanized on either day 7, 14, or 21 following their injection with CFA or saline. All experimental procedures were approved by the Institutional Animal Care and Use Committee (IACUC) (n° 20-08-572) at the American University of Beirut.

### 4.2. Blocking of NMDA Receptors

Thirty-six rats, nine rats per group, were injected in their posterior left knee synovial cavity with ketamine either at a high dose (30 mg/kg) or a low dose (10 mg/kg) one hour before the administration of the inflammatory agent, CFA or saline, on day 1 (Figure 13). Therefore, the four groups, each consisting of nine rats, are: low-dose ketamine inflamed rats (LDKINF), high-dose ketamine inflamed rats (HDKINF), low-dose ketamine control rats (LDKCN), and high-dose ketamine control rats (HDKCN) (Figure 13). This pre-administration aimed to effectively block NMDA receptors and preemptively attenuate the inflammatory response. Additionally, repetitive doses of ketamine were administered on days 7, 14, and 21 (LDK or HDK in Figure 1) to maintain continuous inhibition of NMDA receptors throughout the study duration. Three animals from each group were euthanized at the time points, day 7, 14, and 21.

### 4.3. BrdU Preparation and Administration

The 5-bromo-2′-deoxyuridine (BrdU, Sigma-Aldrich, St. Louis, MO, USA,) was used to identify proliferating cells within living tissues. To prepare the BrdU solution, BrdU powder (Sigma-Aldrich B5002-IG) was accurately weighed and dissolved in warm 0.9% saline. The solution was thoroughly dissolved and subsequently filtered using a 0.2 mm filter unit to ensure purity. Two groups, each of nine rats, were injected with BrdU (66 mg/kg/300 mL/injection, intraperitoneal) 12 h prior to the administration of the inflammatory agent CFA (*n* = 9) or the saline solution (*n* = 9) to maximize the availability of BrdU for incorporation into replicating cells (BrdU CN and, BrdU INF groups, Figure 13). The rats received three BrdU injections at 2 h intervals the day 1.

### 4.4. Joint Circumference Measurement

The circumference of the injected (left) and contralateral (right) knee joints was measured as an indicator of inflammation resulting from knee synovial cavity injection of saline or CFA using a flexible tape measure wrapped mid-patella around the center of the knee joint, as reported in the literature [52].

### 4.5. Nociceptive Behavioral Testing

#### 4.5.1. Motor Behavioral Test

Animals were placed on the Rotarod machine set at a fixed speed of 5 rpm. Each rat was tested five times before the induction of inflammation and on days 7, 14, and 21, with each trial lasting for 300 s and separated by at least a 10 min inter-trial period. The latency to fall off the rotating rod was recorded.

#### 4.5.2. Heat Hyperalgesia Test

A heat hyperalgesia test was done on days 7, 14, and 21 in CN and INF rats by measuring the foot withdrawal latency to a radiant heat applied to the plantar surface of the hind paw [53]. Approximately 15 min prior to testing, each rat was placed individually in a clear plastic cage placed atop an elevated 3-mm thick glass plate to accommodate. The height of the glass plate was adjusted so that the light-based heat stimulus applied to the plantar surface of the normal foot evoked a withdrawal response after approximately 20 s. To avoid adapting limb withdrawal, heat stimuli were applied 5 times with a 5 min resting period between trials. The heat stimulus consisted of a focused beam of light produced by a 50 W halogen light bulb encased in a vented container. The withdrawal latency was defined as the elapsed time, in seconds, from stimulus onset to paw withdrawal; a cut-off of 20 s was imposed to prevent tissue damage.

#### 4.5.3. Mechanical Allodynia Test

On days 7, 14, and 21, CN and INF rats were tested for the paw withdrawal frequency to a non-noxious stimulus determined by applying a von Frey filament with a bending force of 2 g to the plantar surface of the hind paw [54]. The tip of the filament was applied perpendicularly to the medial plantar surface from below the mesh grid until a withdrawal was observed. An increased response indicated the development of mechanical allodynia. Before testing, all rats were placed in a transparent chamber on a metal wire mesh floor and left for a 30 min acclimatization period. Testing started by poking the paw 5 successive times with the filament. Five trials were used, separated by 5 min intervals. Measurements were averaged for each animal, and the responses of all rat groups’ left and right paws were recorded.

#### 4.5.4. Mechanical Hyperalgesia Test

On days 7, 14, and 21, CN and INF rats were tested for the paw withdrawal frequency to a noxious stimulus determined by applying a von Frey filament with a bending force of 15 g to the plantar surface of the hind paw [55]. Testing started by poking the plantar surface 5 successive times with the 15 g filament until the animal stimulated a behavioral response. Five trials were conducted, separated by 5 min time interval. Measurements were averaged for each animal, and the responses of both the left and the right paws of all rat groups were recorded.

### 4.6. Cervical Dislocation and Sample Collection

The cervical dislocation procedure was performed on the different groups: group CN (*n* = 15), group INF (*n* = 15), and the ketamine groups (*n* = 36) were subjected to the procedure at designated time points (Figure 1). Prior to the dislocation, all rats were deeply anesthetized using ketamine (ketalar: 50 mg/kg) and Xyla (xylazine; 12 mg/kg, Interchemie Werken “De Adelaar” B.V., Castenray, The Netherlands). Subsequently, spinal cord tissues were collected from all rats. The lumbar section of the spinal cord was dissected into two segments. The first segment was promptly frozen in liquid nitrogen and stored at −80 °C for subsequent Western blot analysis, while the other segment was processed into paraffin-embedded blocks for immunofluorescence experiments. Synovial membranes were collected only from CN and INF group rats and stored at −80 °C for subsequent Western blot analysis.

### 4.7. Animal Perfusion and Sample Collection

Rats that received BrdU injections (*n* = 18) were deeply anesthetized using ketamine (ketalar: 50 mg/kg) and Xyla (xylazine: 12 mg/kg) at days 7, 14, and 21 following injection with CFA or saline, and synovial membranes were subsequently collected. Following this, the rats underwent transcardial perfusion with 0.9% saline solution, followed by perfusion with 4% paraformaldehyde dissolved in 0.1 M phosphate buffer (PB, pH 7.4). After the spinal cords were carefully removed, the tissues were post-fixed overnight in paraformaldehyde and then transferred to a solution of 30% sucrose in Phosphate-Buffered Saline (PBS) (Sigma-Aldrich, St. Louis, MO, USA) solution at 4 °C for 48 h or until processing time.

### 4.8. Tissue Processing

Spinal cord sections spanning the L3-L4-L5 segments were meticulously sliced at 40 μm thickness utilizing a freezing microtome. A total of 180 slices were systematically collected in wells containing a solution of Sodium Azide (0.38 g/100 mL PBS). The collection of spinal cord sections followed a systematic sampling approach based on the fractionator method to ensure unbiased stereology [56]. In accordance with this method, sections were distributed across six wells, each containing a predetermined number of slices designated to a specific region of the spinal cord (L3, L4, L5). The arrangement involved placing the 1st section in the first well, the 2nd section in the second well, and so forth, until the 6th section was deposited in the sixth well. Subsequently, the sequence repeated, ensuring that the 7th section was positioned in the first well, thus maintaining a consistent interval of 300 mm between the 1st and 7th sections. This systematic approach ensured that each well represented a random depiction of every topographic area within the spinal cord [56]. Finally, a single well was randomly selected from each region for immunofluorescent staining to detect neurogenesis.

### 4.9. Immunofluorescence Detection and Quantification of BrdU

Tissues were washed 3 times with 1× PBS for 5 min each and treated with 2 N HCL for 30 min at 37 °C to allow DNA strands to open and expose the integrated BrdU. Tissues were washed 1 time with 1× PBS for 5 min and then neutralized with borate buffer for 10 min at room temperature. Next, tissues were washed 3 times with 1× PBS for 5 min each, and blocked for 1 h at 4 °C with a 10% blocking solution (Sigma-Aldrich, St. Louis, MO, USA) (1 g BSA, 1 mL Normal goat serum (NGS), and 10 microliter triton X in 10 mL PBS), after which they were incubated overnight with anti-BrdU (See Table 1) diluted in a 3% blocking solution (0.3 g BSA, 300 μL NGS, and 10 μL X in 10 mL PBS). The tissues were then washed 3 times with 1× PBS for 5 min each and incubated with secondary antibody (goat-anti mouse 568, dilution 1:200, abx142334) for 2 h at room temperature and placed on a shaker. Again, tissues were washed 3 times with 1× PBS for 5 min each and incubated with a primary antibody to the neuronal marker overnight anti-NeuN (Table 1). Tissues were then washed 3 times with PBS, 5 min each, and incubated with secondary antibody (goat-anti rabbit 488, dilution 1:250, abx142326) for 2 h and placed on a shaker, before the 2 h end by 10 min. Nuclei were stained with DAPI (dilution 1:10,000 in PBS, DAPI D1306, Thermo Fisher, Waltham, MA, USA), followed by 3 washings for 5 min each with PBS. Lastly, tissues were mounted on slides and covered after the application of an anti-fading mounting medium. Using a laser scanning confocal microscope (Zeiss LSM 710, Jena, Germany), BrdU-positive cells were visualized using the 40X-oil objective and counted to quantify the number of stem/progenitor cells at each time point. Sections were taken from a representative well. The number of BrdU-positive cells was multiplied by 6 to estimate the full count in each region of the spinal cord. These numbers were then added to calculate the total number of positive cells in the whole spinal cord. The total number of BrdU-positive cells in all sections taken from the control group was compared to those taken under inflammatory conditions at different time points.

### 4.10. Immunohistochemistry

Immunohistochemistry was performed on 5 μm thick sections of paraffin-embedded spinal tissue, obtained with a cutting microtome. The sections were dewaxed in xylene for 30 min at 60 °C, rehydrated at room temperature, by sequential immersion in a graded series of alcohols, and transferred into water for 5 min. Subsequently, the serial sections were incubated in antigen unmasking solution (10 mM tri-sodium citrate, 0.05% Tween-20, pH 6) for 8 min at 95 °C and, afterward, immersed in acetone at −20 °C for 5 min to prevent the detachment of the sections from the slide. All subsequent reactions were conducted at room temperature. After a wash with PBS for 5 min, sections were immunostained using Histostain^®^-Plus 3rd Gen IHC Detection Kit (Thermo Fisher Scientific Cat. No. 85-9073, Waltham, MA, USA), which utilized the labeled biotin-streptavidin methodology. The antibody used was mouse monoclonal CD68 (Table 1). Appropriate positive and negative (isotype) controls were run concurrently. Nuclear counterstaining was carried out using hematoxylin (Hematoxylin aqueous formula, N. Cat. S2020, DAKO, Carpinteria, CA, USA). Finally, the slides were prepared for observation with coverslips with an aqueous mounting solution, PermaFluor Mountant (Thermo Fisher Scientific, Waltham, MA, USA). The observation of the sections was performed with an Axioscope 5/7 KMA optical microscope (Carl Zeiss, Milan, Italy) equipped with an Axiocam 208 color digital camera (Carl Zeiss, Milan, Italy). All observations were performed by two independent observers who evaluated the percentage of immunopositivity at two separate times and performed a quantitative analysis. The percentage of positive cells was calculated in a high-power field (HPF) (magnification 400×) and repeated for 10 HPFs. The arithmetic mean ± standard deviation of evaluation was used for statistical analysis. The final percentage value for each case was the arithmetic mean of the 10 values obtained, and this arithmetic mean of counts was used for statistical analysis.

### 4.11. Immunofluorescence and Confocal Microscopy

For IF, deparaffinized 5–10 μm sections were incubated in the “antigen retrieving solution” (10 mM tri-sodium citrate, 0.05% Tween-20) for 8 min at 75 °C, and treated with a blocking solution (3% BSA in PBS) for 30 min. Next, the primary antibodies indicated in Table 1 were applied in different sections (anti-Hsp27, anti-Hsp60, anti-Hsp70, anti-Hsp90, or anti-GFAP), and the sections were incubated in a humidified chamber at 4 °C overnight. Then, we incubated the sections for 1 h at 25 °C with the corresponding conjugated secondary antibody (goat anti-rabbit IgG Atto 647 N, 40839-1ML-F, Sigma-Aldrich, St. Louis, MI, USA; goat anti-mouse IgG Atto 488N, 62197-1ML-F, Sigma-Aldrich, St. Louis, MI, USA; donkey anti-goat IgG-FITC, SAB4600032; goat anti-rabbit IgG–TRITC antibody, T5268, Sigma-Aldrich, St. Louis, MI, USA; goat anti-mouse IgG-FITC antibody, F5897, Sigma-Aldrich, St. Louis, MI, USA). Nuclei were stained with DAPI (dilution 1:1000). The slides were bathed with PBS drops and mounted on coverslips. The images were captured using a Leica Confocal Microscope TCS SP8 (Leica Microsystems, Heidelberg, Germany). Staining intensity for Hsp27, Hsp60, and Hsp90 was expressed as the mean pixel intensity (PI) normalized to the CSA (cross-sectional area expressed in pixels) using the software Leica application suite advanced fluorescence software, as previously described [57,58,59,60].

### 4.12. Western Blot

Spinal cord tissues collected from rats of the CN, CFA, and ketamine groups, which underwent neck dislocation, alongside synovial membranes obtained before the perfusion of the BrdU-treated groups at designated time points, were lysed using RIPA buffer. The RIPA buffer composition included 0.1% sodium dodecyl sulfate (SDS), 0.5% sodium deoxycholate, 10% Triton X-100, 25 mM HEPES, 500 mM DTT, 1.5 mM magnesium chloride, 300 mM sodium chloride, 200 μM EDTA, and 10 mM NaF, along with 2% protease inhibitors. Subsequently, the lysates underwent centrifugation at 13,500 rpm for 20 min at 4 °C, and the protein concentration in the supernatants was determined using the Bradford Protein Assay.

For immunoblotting, 60 μg of proteins were separated on a 12–15% polyacrylamide gel Electrophoresis (Bio-Rad Laboratory, Hercules, CA, USA) and then transferred onto nitrocellulose membranes (Bio-Rad Laboratory, Hercules, CA, USA). Precision Plus Protein™ Kaleidoscope™ (Bio-Rad Laboratory, Hercules, CA, USA) was used to estimate the molecular weight of proteins.

These membranes were blocked with 5% BSA in Tris-buffered saline and then incubated overnight with antibodies against Hsp90, Hsp70, Hsp60, Hsp27, or GFAP (refer to Table 1 for details). The primary antibodies were detected using horseradish peroxidase-conjugated IgG (rabbit IgG, 1:20,000, GTX26795, Gene Tex, Irvine, CA, USA; mouse IgG, 1:10,000, AP124P, Chemicon International, Temecula, CA, USA; goat IgG, 1:10,000, Sigma-Aldrich, St. Louis, MI, USA). Bands were visualized using enhanced chemiluminescence, and densitometric analysis was performed using ImageJ 1.41 software (National Institute of Health, Bethesda, MD, USA).

### 4.13. Statistical Analysis

Statistical analyses were performed using the GraphPad Prism 8 software. Data were presented as means ± standard errors of the means (S.E.M) in the behavioral experiments, while they were represented as means ± standard deviation (SD) in the rest of the experiments. In normally distributed populations, significant changes in latency and withdrawal frequencies within groups over time were determined with one-way repeated measures analysis of variance (ANOVA) of the raw data, followed by Bonferroni for post hoc analysis. In non-normally distributed populations, significant changes were calculated using Friedman’s analysis of variance on ranks. A comparison of paw withdrawal latency to heat and mechanical stimulation before and after induction of inflammation at all testing times was performed using paired *t*-tests. Differences are considered significant at *p* < 0.05. In the other experiments, data are presented as standard deviation ± standard error of the standard deviation.

## 5. Conclusions

In this study, we show that in the RA rat model, the induction of inflammation does not lead to HSP variation in the synovial membrane, while in the spinal cord, there is an observed increase of Hsp90 and Hsp70 during the initial stages of the disease, indicating their potential pro-inflammatory roles or their involvement in a negative feedback loop still unknown. Conversely, the decreased expression of Hsp60 in the latter stages suggests its anti-inflammatory role. Although the exact impacts of altered HSP levels during RA onset on adult neurogenesis remain incompletely understood, we speculate that it may influence neuroplasticity and cellular reconfiguration, potentially correlated with changes in the heat shock protein levels. Furthermore, our findings reveal a reduction in pain perception and a decrease in Hsp90 and Hsp70 levels in the spinal cord of RA rats treated with ketamine, both at low and high doses, indicating a potential therapeutic effect. As previously discussed, ketamine is reported to have anti-inflammatory effects through modulation of CNS cells; however, NMDA receptors may also play pivotal roles in the long-term progression of rheumatoid arthritis, potentially inducing the release of HSPs involved in the inflammatory processes. Despite the limitations of this research, such as the number of rats evaluated, our study underscores the necessity for further investigation into the roles of HSPs in RA and suggests their modulation as a possible therapeutic approach.

## Figures and Tables

**Table 1:** The primary antibodies used for immunostaining and Western blot experiments.

Primary Antibody	Animal Host	Distributor	WB	IF	IHC
Glyceraldehyde 3-phosphate dehydrogenase (GAPDH)	Rabbit	LOT-3557958, Rabbit Polyclonal EMD Millipore Corp., Burlington, MA, USA	1:1000	**-**	**-**
Heat Shock Protein 90 (Hsp90)	Rabbit	Ab-13495, Rabbit Monoclonal Abcam, Cambridge, UK	1:1000	1:50	**-**
Heat Shock Protein 70 (Hsp70)	Mouse	Ab-2787, Mouse Monoclonal Abcam, Cambridge, UK	1:1000	1:50	**-**
Heat Shock Protein 60 (Hsp60)	Mouse	Ab-13532, Mouse Monoclonal Abcam, Cambridge, UK	1:1000	1:50	**-**
Heat Shock Protein 27 (Hsp27)	Goat	Sc-1048, Goat Polyclonal Santa Cruz Biotechnology, Dallas, TX, USA	1:1000	1:50	**-**
Glial Fibrillary Acidic Protein (GFAP)	Mouse	Sc-33673, Mouse Monoclonal Santa Cruz Biotechnology, Dallas, TX, USA	1:1000	1:50	**-**
Bromodeoxyuridine (BrdU)	Mouse	Sc-32323, Mouse Monoclonal Santa Cruz Biotechnology, Dallas, TX, USA	**-**	1:100	**-**
Neuronal Nuclear Antigen (NeuN)	Rabbit	R-A22113, Rabbit Monoclonal Neuromics, Edina, MN, USA	**-**	1:500	**-**
Cluster of Differentiation 68 (CD68)	Mouse	Sc-20060, Mouse Monoclonal Santa Cruz Biotechnology, Dallas, TX, USA	**-**	**-**	1:150

## Data Availability

The datasets generated during and/or analyzed during the current study are contained within the article and Supplementary Material.

## Supplementary Materials

The following supporting information can be downloaded at: https://www.mdpi.com/article/10.3390/ijms26199743/s1..
