# Supplementary material for: Exploring the Role of Heat Shock Proteins in Neuroimmune Modulation in Rheumatoid Arthritis: Insights from a Rat Model
Source: Int J Mol Sci. 2025 Oct 7;26(19):9743. doi: 10.3390/ijms26199743 (PMC12525430; doi:10.3390/ijms26199743)
Supplement: Supplementary file 1 [file ijms-26-09743-s001.zip › ijms-3889488-supplementary.pdf]

Research Article

The potential role of heat shock proteins in the spinal cord of rheumatoid arthritis rat model

Malak Fouani [1], Federica Scalia [2,3], Giuseppe Donato Mangano [3], Francesca Rappa [2], Wassim Abou-Kheir [4,†], Angelo Leone [2], Nada Lawand [5,†], and Rosario Barone [2,*]

[1] Department of Neurology, Duke University, Durham, NC, USA
[2] Department of Biomedicine, Neurosciences and Advanced Diagnostics, University of Palermo, Palermo, Italy
[3] Department of Medicine and Surgery, Kore University of Enna, Enna, Italy
[4] Department of Anatomy, Cell Biology and Physiological Sciences, Faculty of Medicine, American University of Beirut, Beirut, Lebanon
[5] Ivy Tech Community College, Indianapolis, IN, USA
* Correspondence: RB rosario.barone@unipa.it
† Co-senior Authors.

Supplementary Material.

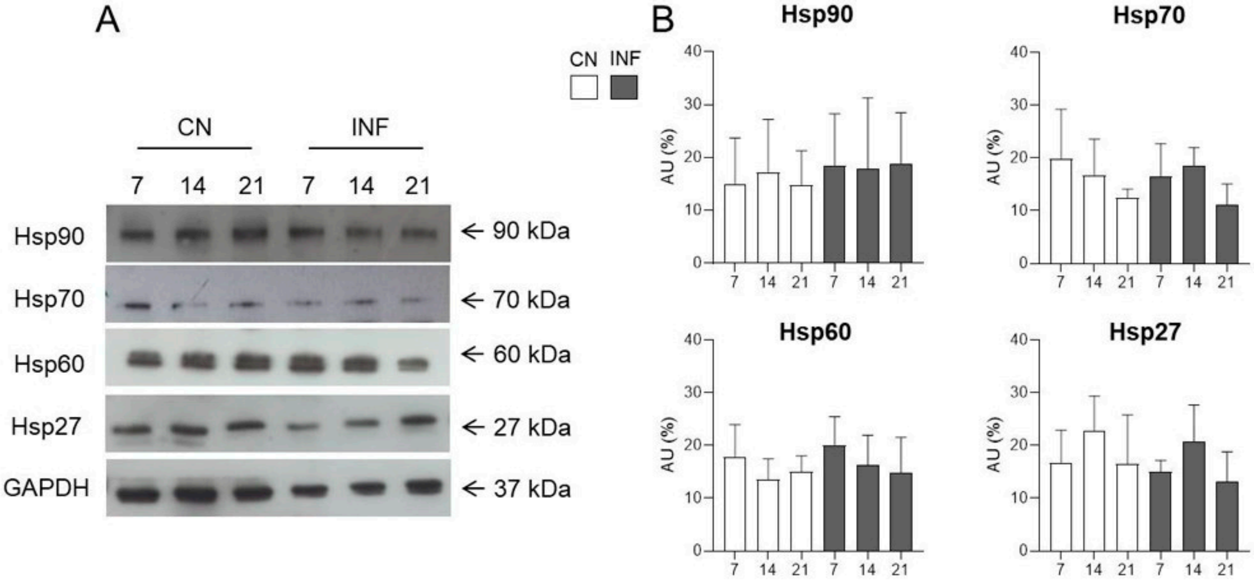

Figure S1. Protein expression levels of Hsp90, Hsp70, Hsp60, and Hsp27 in the synovial members of inflamed and control rat groups

Rats injected in their left posterior knee joint with CFA are the inflamed group (INF) (*n*=12), and ones injected with saline are the control group (CN) (*n*=12). Representative Western blotting of Hsp90, Hsp70, Hsp60, and Hsp27 from the tissue of the two groups is shown in (A), and relative expression levels are observed in (B). GAPDH was used as loading control. Data are presented as the mean ±SD.

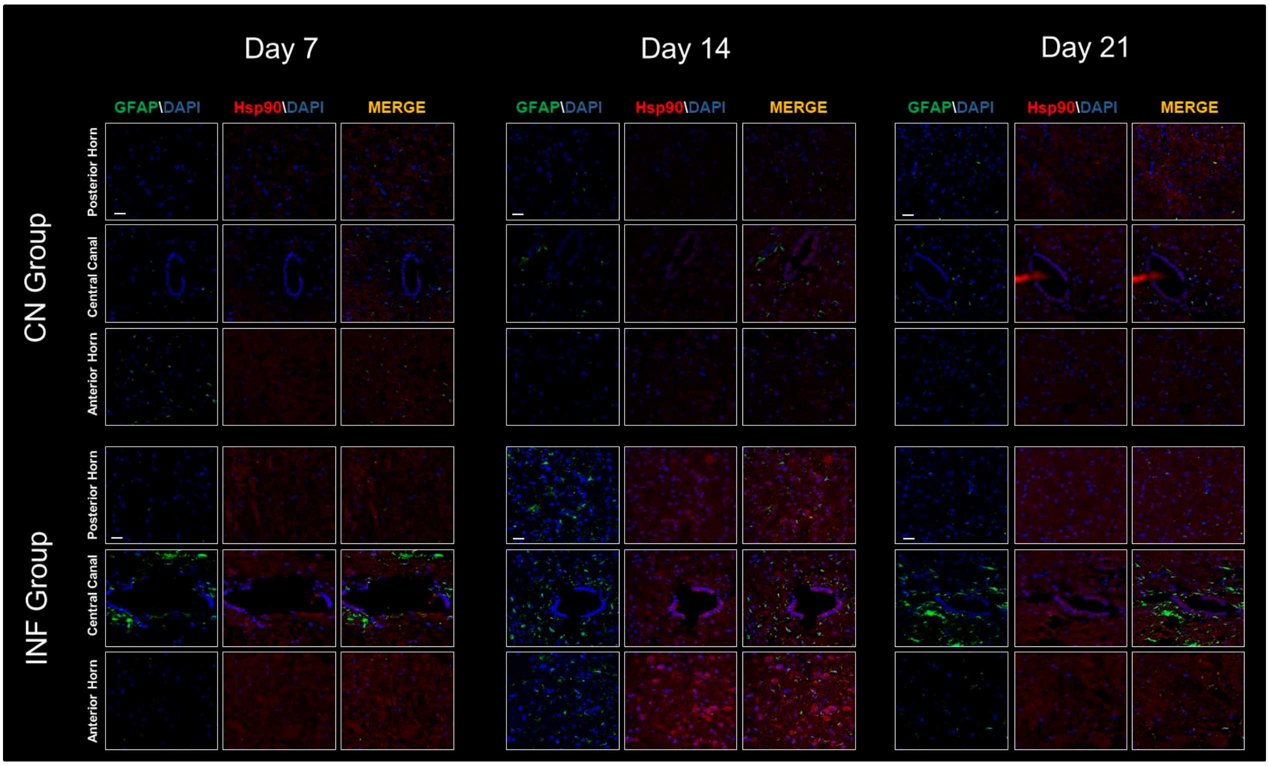

Figure S2A. Double Immunofluorescence of glial fibrillary acidic protein (GFAP) with Hsp90 in spinal cord tissues of control and inflamed groups of rats.

Double Immunofluorescence for glial fibrillary acidic protein (GFAP) (green) and Hsp90 (red) was conducted on the sections of the posterior horn, central canal, and anterior horn of the spinal cords from inflamed (INF) and control (CN) rats, on days 7, 14, and 21. Nuclei have been stained with DAPI (blue signal). Bar = 25 μm.

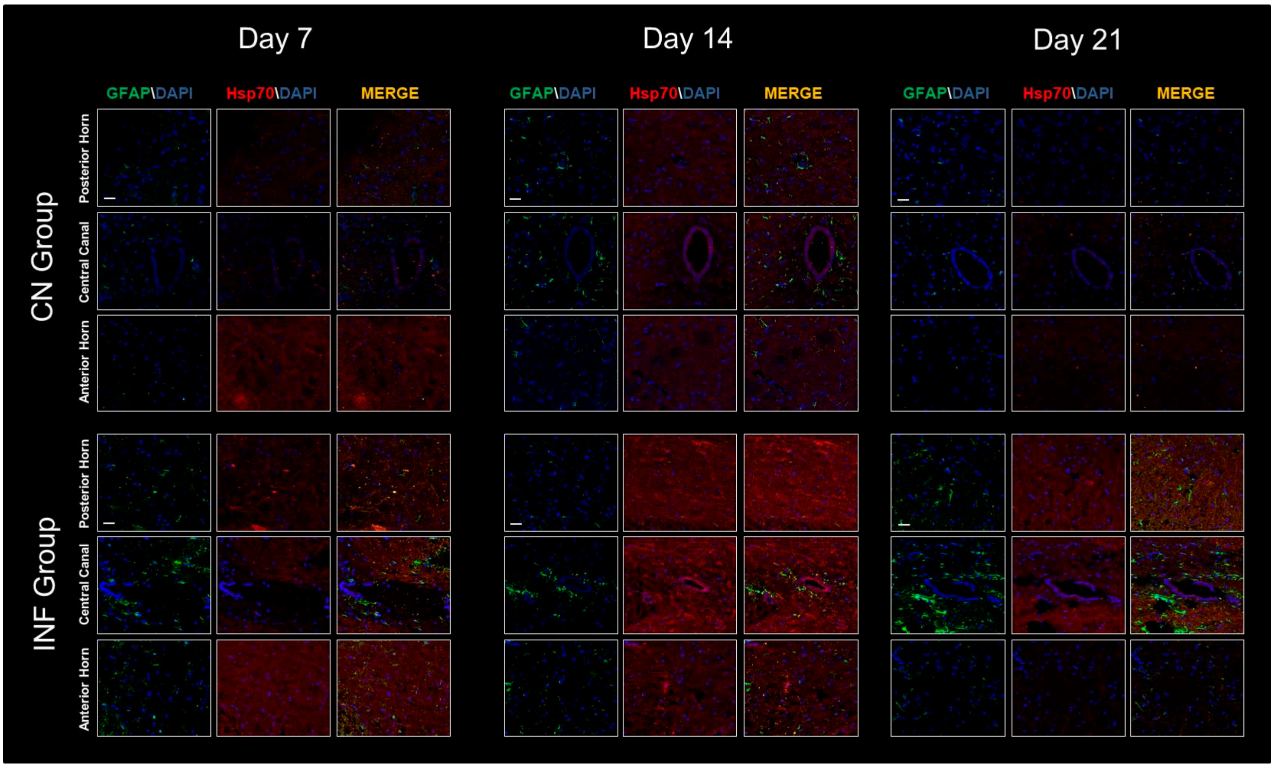

Figure S2B. Double Immunofluorescence of glial fibrillary acidic protein (GFAP) with Hsp70 in spinal cord tissues of control and inflamed groups of rats.

Double Immunofluorescence for glial fibrillary acidic protein (GFAP) (green) and Hsp70 (red) was conducted on the sections of the posterior horn, central canal, and anterior horn of the spinal cords from inflamed (INF) and control (CN)

rats, on days 7, 14, and 21. Nuclei have been stained with DAPI (blue signal). Bar = 25 μm.

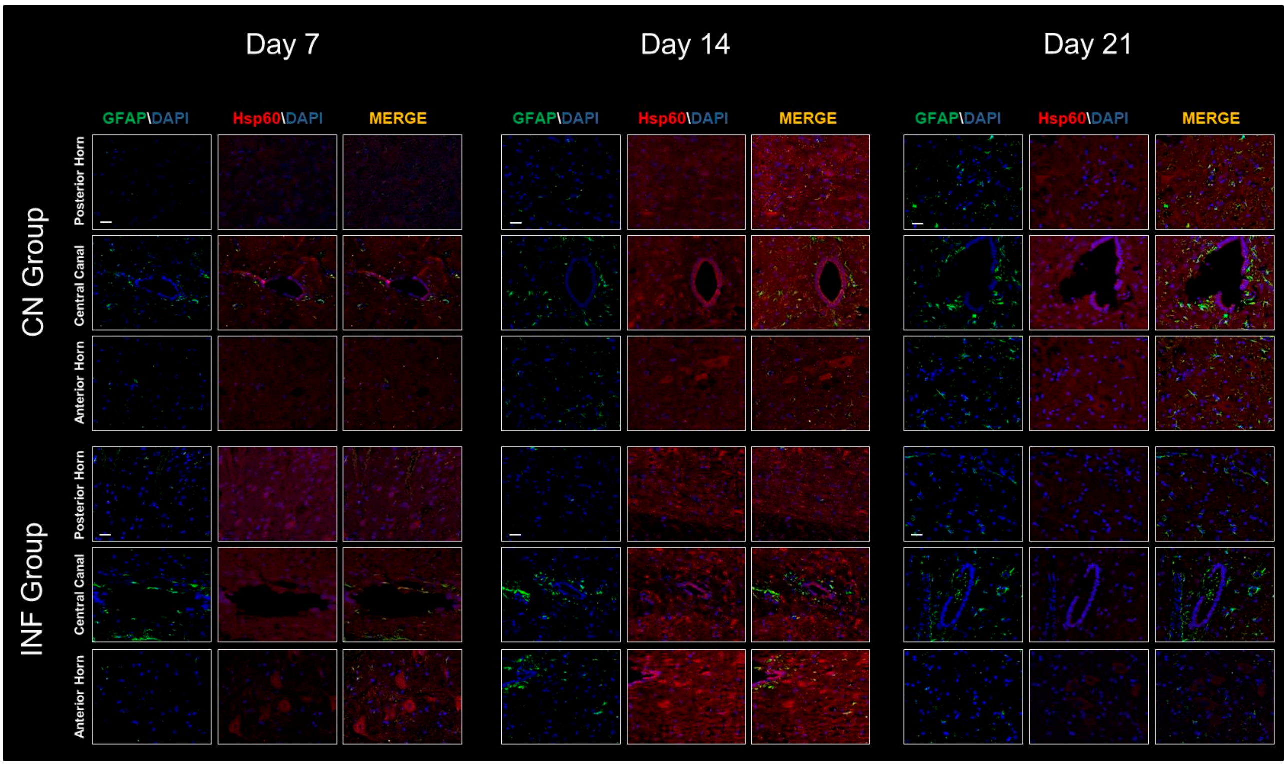

Figure S2C. Double Immunofluorescence of glial fibrillary acidic protein (GFAP) with Hsp60 in spinal cord tissues of control and inflamed groups of rats.

Double Immunofluorescence for glial fibrillary acidic protein (GFAP) (green) and Hsp60 (red) was conducted on the sections of the posterior horn, central canal, and anterior horn of the spinal cords from inflamed (INF) and control (CN) rats, on days 7, 14, and 21. Nuclei have been stained with DAPI (blue signal). Bar = 25 μm.

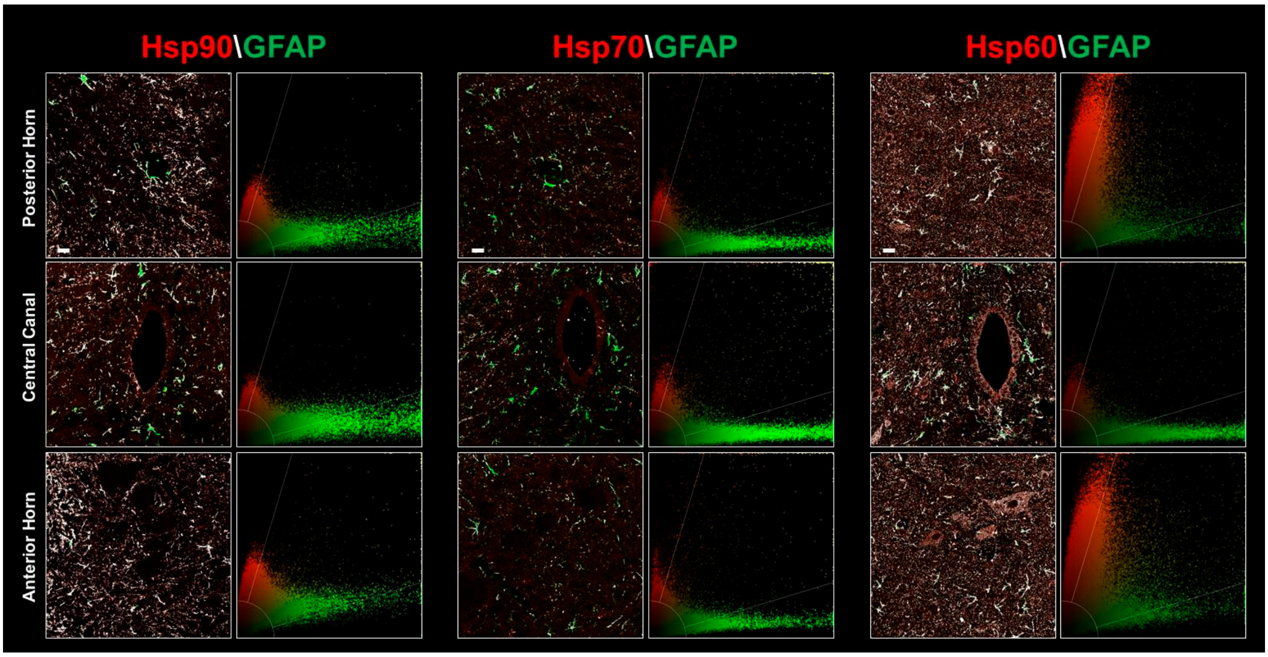

Figure S3A. Colocalization of glial fibrillary acidic protein (GFAP) with the Hsp90, Hsp70, and Hsp60 in rat spinal cord tissues.

Representative images in the different regions of the spinal cord tissues (Anterior horn, Central Canal, and Posterior horn) of a rat saline-injected on day 14, are shown. The panel shows HSPs (90, 70, and 60) with red signal and GFAP with green signal. Colocalization is represented by the white color in the tissue slides and by the related graph on the right: the greater the localization of the green and red pixels in the center of the graph, the greater the colocalization of the two considered proteins. Bar = 25 μm.

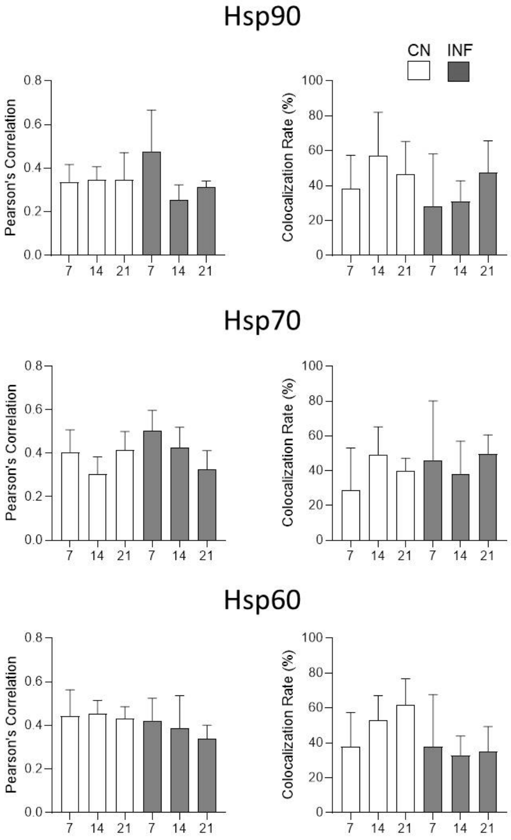

Figure S3B. Pearson's correlation and colocalization rate of GFAP and the Hsp90, Hsp70, and Hsp60 in rat spinal cord tissues from control and inflamed rats.

Rats injected in their left posterior knee joint with CFA are the inflamed group (INF), and ones injected with saline are the control group (CN). The colocalization was analyzed in the different random regions of the spinal cord at each time point. Data are presented as the mean ±SD.
